# Green Extraction of Microcrystalline Cellulose from Rice Straw and Determination of Its Reinforcing Capacity in PHBV Films

**DOI:** 10.3390/polym18121489

**Published:** 2026-06-13

**Authors:** Pedro Augusto Vieira de Freitas, Chelo González-Martínez, Amparo Chiralt

**Affiliations:** Institute of Food Engineering FoodUPV, Polytechnic University of Valencia, 46022 Valencia, Spain; cgonza@tal.upv.es (C.G.-M.); dchiralt@tal.upv.es (A.C.)

**Keywords:** high-shear hydrolysis, subcritical water extraction, poly(3-hydroxybutyrate-co-3-hydroxyvalerate), cellulose fibres, crystallinity

## Abstract

Rice straw is a highly produced agricultural waste with a high cellulose content, which can be used as a cellulose source. Nevertheless, more sustainable extraction and purification strategies are needed to reduce the consumption of chemicals during the production of cellulose-derived materials. In this way, an integrated method based on subcritical water extraction and bleaching with hydrogen peroxide was used for isolating cellulose from rice straw. The cellulose fibres obtained were converted into microcrystalline cellulose (MCC) by applying acid hydrolysis with HCl 2N at 60 °C to reduce the fibre amorphous fraction. High cellulose purity (86%) and crystallinity (67%) were obtained in the isolated fibres. The influence of high-shear homogenisation (12,000 rpm) during hydrolysis was analysed, compared to mild stirring (350 rpm) at different times (30 and 60 min). High-shear homogenisation greatly accelerated the hydrolysis process of the amorphous fraction of the fibres, contributing to the reduction in particle size (to about 10 µm), defibration, increased crystallinity (70–72%), and shorter cellulose chains (92,400–61,600 g/mol) for a given treatment time. After 30–60 min of treatment, the resulting MCCs exhibited properties within the range reported for commercial AVICEL, with greater reinforcing performance in PHBV films. These MCCs resulted in lower water vapour permeability, while improved oxygen barrier properties were mainly observed for those obtained under high-shear hydrolysis conditions.

## 1. Introduction

Microcrystalline cellulose (MCC) is widely used in various sectors, such as the food, pharmaceutical, medical and cosmetics industries, and its demand is growing as an alternative to non-renewable fossil-based materials. MCC is of great interest due to its renewable origin, safety, economic value, biodegradability, high mechanical properties, large specific surface area and biocompatibility. Therefore, new sources and isolation processes for cellulose, as well as new treatments to obtain MCC, are being studied to meet the growing demand for the industrial-scale production of new types of MCC-based materials [1,2,3]. Cellulose has been mainly extracted from wood and cotton. Nevertheless, to meet the demand for raw materials, the use of renewable resources, such as lignocellulosic residues, must be promoted to obtain this natural polymer. Lignocellulosic residues from agricultural activities are available in large quantities and at low cost, with cellulose content ranging from 40 to 78% [4]. Thus, these represent a renewable cellulose source and a primary raw material for producing MCC or nanocellulose. Likewise, the search for more sustainable processes to obtain cellulose derivatives with adequate properties for different applications is of great interest in order to found processes that are economically viable and environmentally friendly [5].

The use of lignocellulosic waste as a cellulose source requires the analysis of its composition and typical properties, such as crystallinity and thermal stability. The final chemical composition of cellulosic materials depends on their origin and the processing method [6]. The production of nanocellulose and MCC involves two main processes: the extraction and purification of cellulose from the lignocellulosic source, followed by a hydrolysis treatment that reduces the amorphous phase of cellulose and enhances its crystallinity. Cellulose extraction can be achieved through different approaches, including ultrasound-assisted extraction, alkaline treatments such as soda pulping, or combinations of physicochemical processes [7]. Subsequent purification stages are commonly applied to remove residual lignin and hemicelluloses, with hydrogen peroxide bleaching being one of the most commonly used methods [8]. Both extraction/purification and hydrolysis affect the morphology, crystallinity, and thermal stability of the resulting cellulosic material, which may ultimately influence its performance in different applications.

The crystallinity of MCC is considered high, ranging between 50 and 80% [9,10]. Industrial production of MCC is usually carried out by hydrolysis of bleached cellulose with dilute mineral acids (mainly HCl or H_2_SO_4_), using cotton and wood as cellulose sources. However, as recently revised by Ventura-Cruz et al. [6], MCC has also been obtained and characterized from many sources, such as oil palm fronds, date seeds, pomelo peel, roselle fibres, olive stems, *Posidonia oceanica*, corn cobs, sugarcane bagasse, coconut husk fibres, agave tequilana, barley waste, forest residues, cassava root bagasse, corn straw and residual rose stems [6].

Acid hydrolysis is the most usual and convenient process to obtain MCC after the bleaching step of isolated cellulose fibres. This treatment reduces particle size, increases crystallinity, and improves mechanical properties of cellulose [11]. The acid attacks the amorphous phase of the fibres, while the crystalline ones remain mostly insoluble in acids [12]. Apart from H_2_SO_4_, HCl is the most widely used acid to obtain MCC. Katakojwala and Mohan [5] used H_2_O_2_ at different concentrations and temperatures to depolymerise bleached cellulose fibres obtained from sugarcane bagasse. H_2_O_2_ promoted hydrolysis and cellulose chain-breaking, yielding small glucose chains and MCC while improving bleaching. Other authors [13] have also used different concentrations of alkaline H_2_O_2_ to produce MCC from oil palm leaves, which affected the final crystallinity and quality of the MCC. The final MCC properties depend on the origin of fibres, extraction and bleaching treatments, and hydrolysis conditions, such as the concentration of the hydrolytic agent, temperature, and contact time, with the latter being considered the most important factor during the reaction [14].

Mechanical treatments have also been applied in the different steps of cellulose purification [15] or modification, with positive effects on fibre disaggregation and depolymerisation. Bandera et al. [16] obtained cellulose whisker-like materials by mechanical treatment of commercially available MCC. High-shear homogenisation of the MCC aqueous dispersions with a high solid content allowed for the production of cellulose particles with greatly reduced dimensions and a similar reinforcing effect to that of cellulose whiskers. Likewise, Borsoi et al. [17] obtained cellulose nanowhiskers by mechanical grinding in a supermass colloid. Mechanical pretreatment of Kraft eucalyptus pulp facilitated acid hydrolysis, thereby enhancing the yields of nanocellulose and MCC [18]. It is necessary to optimise the different stages of the MCC production process, taking into account the chemicals used to extract, bleach and hydrolyse the fibres, as well as process variables such as temperature, concentration and reaction times, to improve its economic viability, sustainability and scalability.

It is well known that rice is the most widely grown cereal in the world, with about 100 million tons produced annually [19]. Its production generates 45% of its weight in rice straw, which is therefore a very abundant non-woody raw material with a high cellulose content (near 40%). In fact, cellulose isolated from rice straw can be used as low-cost feedstock for producing value-added products such as MCC, methylcellulose, or cellulose acetate after the complete removal of hemicellulose, lignin, and ash. Ibrahim et al. [20] obtained MCC from rice straw by applying a first pulping treatment (alkaline–acid or acid–alkaline), followed by bleaching with sodium hypochlorite. MCC was then obtained from isolated cellulose via enzymatic treatment, resulting in cellulose with different lengths and diameters depending on the treatment time. Fan et al. [21] also obtained MCC from crude cellulose isolated from rice straw via partial hydrolysis, using microwave heating in both the isolation (acid and alkali treatments) and hydrolysis processes. The cellulose content of MCC was 93.6 wt %, with no significant differences in cellulose characteristics between the microwave-assisted and traditional heating methods. Wistara & Fatriasari [22] used a soda pulping process, followed by bleaching with H_2_O_2_ to obtain cellulose from rice straw. The resulting cellulose was hydrolysed with HCl and ground to achieve the desired form of MCC, which was used for papermaking with unbleached rice straw pulp.

Recently, Freitas et al. [8] described a process for reducing chemical use to obtain cellulose fibres from rice straw, combining subcritical water extraction (SWE) to remove a large proportion of non-cellulosic compounds, followed by a chlorine-free bleaching step with alkaline hydrogen peroxide. Specifically, SWE at 180 °C, followed by four bleaching cycles with H_2_O_2_ (4%, at pH 12, for 1 h), yields high-purity cellulose fibres (86%) and a good yield relative to the raw material (34.9%), making it an environmentally friendly and more sustainable alternative for isolating cellulose fibres from rice straw. SWE allows for selectively extracting many organic compounds from plant matrices due to the unique and useful characteristic of subcritical water, whose polarity dramatically decreases with increasing temperature, behaving similarly to methanol or ethanol [23]. Likewise, the elevated pressure can favour the extraction by forcing water to penetrate into the matrix pores, increasing the contact surface. Subcritical water technology is also an efficient green technique for both extraction and hydrolysis of protein and other fractions (lipid, carbohydrates, and phenolics) due to its hydrolytic capacity [24].

The aim of this study was to produce MCC from rice straw using the more environmentally friendly process described above, isolating cellulose fibres via subcritical water extraction, followed by chlorine-free bleaching with hydrogen peroxide. Furthermore, during subsequent acid hydrolysis, the effect of high shear on MCC production was analysed in comparison with mild agitation. The typical properties of the MCC obtained in the different treatments were analysed, as well as its reinforcing capacity in PHBV films.

## 2. Materials and Methods

### 2.1. Plant Material and Chemicals

RS (*O. sativa* L. variety *J. Sendra*) was collected from L’Albufera paddies (Valencia, Spain). Prior to extraction, the RS was vacuum-dried at 55 ± 2 °C and 0.8 mbar for 16 h, followed by grinding in 3 cycles of 90 s each using a mill machine (model M20, IKA, Satufen, Germany), then sieved to obtain particles of under 0.5 mm. Poly(3-hydroxybutyrate-co-3-hydroxyvalerate (PHBV) was purchased from TianAn Biologic Materials (Ningbo, China) with 2% mol hydroxyvalerate. Hydrochloric acid (HCl, 37%) was purchased from Scharlab S.L. (Sentmenat, Barcelona, Spain). Sodium hydroxide (NaOH), arabinose and glucose were supplied by Sigma-Aldrich (St. Louis, MO, USA). Hydrogen peroxide (H_2_O_2_, 30%), sulphuric acid (H_2_SO_4_, 98%), P_2_O_5_ (98.2%), and sodium carbonate (Na_2_CO_3_) were obtained from Panreac Quimica S.L.U (Castellar del Vallés, Barcelona, Spain). D(+)-xylose and 2 M cupriethylenediamine solution were purchased from Merck KGaA (Darmstadt, Germany).

### 2.2. Obtaining Microcrystalline Cellulose MCC

#### 2.2.1. Isolation of Cellulose Fibres

**Figure 1:**
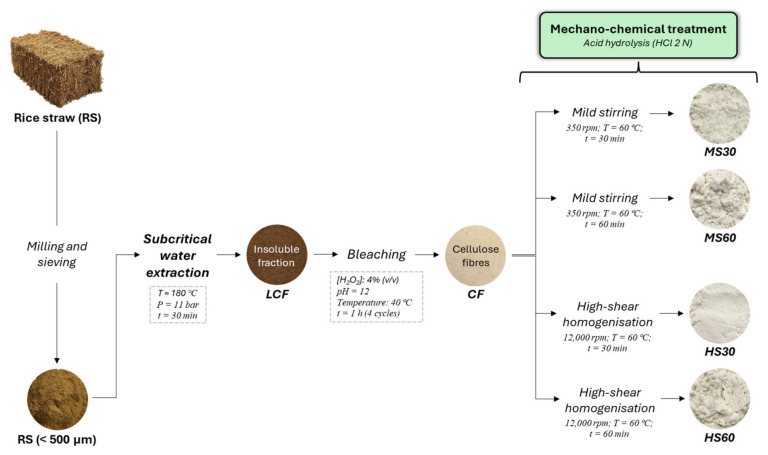
Flow chart and the samples obtained at each process step for obtaining microcrystalline cellulose from rice straw.

The ground RS was subjected to SWE at 180 °C, 11 bar, and 150 rpm for 30 min using a pressure reactor (model 1-T-A-P-CE, 5 L capacity, Amar Equipments PVT. LTD, Mumbai, India) (Figure 1). Afterwards, the plant dispersion was vacuum-filtered, and the insoluble residue from the SWE extraction (LCF) was collected and bleached through 4 bleaching cycles of 1 h each using a bleaching solution composed of hydrogen peroxide (4% (*v*/*v*)) at pH 12 and 40 °C [8]. The obtained bleached cellulose fibres (CF) were conditioned in P_2_O_5_ (~0% RH) at 25 °C until further use to obtain MCC.

#### 2.2.2. Hydrolysis of Bleached Cellulose Fibres

Different mechano-chemical methods were applied to the RS to obtain MCC. The bleached CFs were dispersed in a 2 N hydrochloric acid solution at a solid-to-liquid ratio of 1:25 (*w*/*v*) for the hydrolysis treatment carried out at 60°C. This was carried out using two mechanical treatments: magnetic mild stirring (350 rpm) (MS) at 60 ± 2 °C for 30 (MS30) and 60 min (MS60) and high-shear homogenisation (HS) using a rotor–stator homogeniser (T25 digital ULTRA-TURRAX, IKA Werke GmbH & Co. KG, Staufen, Germany) at 12,000 rpm for 30 (HS30) and 60 min (HS60) (Figure 1). After the corresponding reaction time, each suspension was vacuum-filtered and washed with distilled water to remove residual acid. The resulting MCC samples were dried in an oven at 60 °C for 16 h, ground (three cycles of 1 min) using a milling machine (M20, IKA, Satufen, Germany), and conditioned in P_2_O_5_ (~0% RH) at 25 °C until analyses.

### 2.3. Characterisation of the Cellulosic Fractions

#### 2.3.1. Chemical Composition of the Isolated Fibres

The chemical composition of the cellulosic fractions in terms of cellulose, hemicellulose, and lignin contents was determined according to NREL standard methods [25], following the procedure described by Freitas et al. [7] based on two-step acid hydrolysis with H_2_SO_4_ and subsequent quantification of acid insoluble lignin, and monosaccharides (glucose, xylose and arabinose) were determined by high-performance liquid chromatography (HPLC, Agilent Technologies, model 1120 Compact LC, Waldbronn, Germany) using a HILIC Luna Omega Sugars column (150 × 4.6 mm, 3 μm) coupled to an evaporative light scattering detector (ELSD Agilent Technologies 1200 Series, Waldbronn, Germany). The mobile phase consisted of water (25:75, *v*/*v*) in isocratic mode at a flow rate of 0.8 mL min^−1^. The detector was operated at 40 °C, 3.0 bar N2 pressure, and a gain of 3. The analysis was performed in triplicate for each cellulosic sample.

#### 2.3.2. Colour Properties

The CIEL*a*b colour coordinates of the different cellulosic samples were analysed using a spectrocolourimeter (CM-3600d, Minolta Co., Tokyo, Japan), with a D65 illuminant and a 10° observer. The L* (lightness), a* (red-green), and b* (yellow-blue) values were used to determine the whiteness index (*WI*) of each sample according to Equation (1).

(1) $$WI=100-\sqrt{(100-L^{*}{)}^{2}+a^{*2}+b^{*2}}$$

#### 2.3.3. Morpho-Geometric Analyses

The morphological characteristics of the cellulosic samples were analysed using a field-emission scanning electron microscope (FESEM) equipped with a focused ion beam (AURIGA Compact, Zeiss, Oberkochen, Germany). Before the observation, all samples were coated with platinum using an EM MED020 sputtering system (Leica BioSystems, Barcelona, Spain) for 60 s. The images were obtained at an acceleration voltage of 2 kV.

The particle size distribution of the sonicated (Vibra Cell™ VCX750, 750 W power, 20 kHz frequency, Sonics & Materials, Newton, CT, USA) cellulose aqueous dispersion samples (0.5% (*w*/*v*) for 20 min) was evaluated using a laser diffraction particle size analyser (Mastersizer 3000, Malvern Instruments Ltd., Malvern, UK). The sonicated samples were introduced into the instrument under agitation at 1900 rpm in sufficient quantity to achieve a turbidity of 10%, assuming an absorption coefficient and refractive index of 0.1 and 1.52, respectively. The analysis was performed in 2 repetitions, with 10 replicates per repetition. The particle distribution curves were obtained as the volume fraction versus particle size.

#### 2.3.4. Intrinsic Viscosity, Degree of Polymerisation and Molecular Weight

The intrinsic viscosity ([ƞ]), degree of polymerisation (DP), and molecular weight (Mv) of the different cellulose samples were determined using capillary viscometry, according to the methodologies described in TAPPI T 230 om-08 and Agblevor et al. [26], with modifications. To this, 0.25 g of each conditioned cellulose sample was dispersed in 25 mL of distilled water, with 6 glass beads, for 5 min, using a shaker (KS 130 basic, IKA, Staufen, Germany). Then, 25 mL of the 2 M cupriethylenediamine solution was added to the cellulose dispersion, and the headspace was purged with nitrogen for 1 min before the bottles were closed. The dispersions were kept under agitation for ~60 min until the cellulose was completely dissolved. Afterwards, density (by pycnometry) and kinematic viscosity of each cellulose solution was measured in a calibrated capillary viscometer (capillary diameter 0.4 mm) at 25 °C from the fall time. The measurements were performed in triplicate. Based on the sample viscosity (μ), the intrinsic viscosity ([ƞ], Equation (2)) of the different cellulose samples was calculated, considering its solution concentration (*C*) as well as the degree of polymerisation (*DP*, Equations (3)) [27] and the viscous average molecular weight (*Mv*, Equation (4)) [28].

(2) $$\left[\eta \right]=\frac{{\left[2\left(\mu_{sp}-\ln\mu_{r}\right)\right]}^{1/2}}{C}$$

(3) $$DP=190\times \left[\eta \right]$$

(4) $$M_{v}=162\times DP$$

#### 2.3.5. X-Ray Diffraction (XRD) Analysis

An X-ray diffractometer (AXS/D8 Advance, Bruker, Karlsruhe, Germany), using Kα-Cu radiation (λ = 1.542 Å) and 40 kV, was used to determine the crystallinity of the cellulosic fractions. The spectra of the samples, previously conditioned at 0% RH at 25 °C, were obtained with a scan rate of 0.05°.min^−1^ between 2*θ* of 5° and 40°. The crystallinity index (*CI*) of the samples was determined according to Equation (5) [29].

(5) $$CI\;(\%)=\frac{\left(I_{200}-I_{2\theta =18}\right)}{I_{200}}\times 100$$

where *I*_200_ is the maximum diffraction intensity at 200 (crystalline peak) and *I*_2_*_ϴ__=_*_18°_ is the diffraction intensity at 2*θ* = 18° (amorphous phase valley). The analysis was performed in duplicate for each cellulose sample.

#### 2.3.6. Thermogravimetric Analysis

A thermogravimetric analyser (TGA 1 Stare System analyser, Mettler-Toledo, Greifensee, Switzerland) was used to evaluate the thermal stability of the different cellulosic fractions. About 3–5 mg of each conditioned sample (P_2_O_5_ for 1 week at 25 °C) was placed in an alumina pan and heated from 25 to 700 °C at 10 °C.min^−1^ under a constant nitrogen flow (10 mL·min^−1^). The TGA and the derivative (DTGA) curves were analysed with the STAR^e^ Evaluation Software (Mettler-Toledo, Greifensee, Switzerland) to determine the initial (T_on_) and maximum thermodegradation rate (T_p_) temperatures, as well as the percentage of mass loss at each thermal degradation step.

#### 2.3.7. Fourier-Transform Infrared Spectroscopy (FTIR)

The vibrational profile of the functional groups present in the cellulose samples was evaluated using an FTIR spectrometer (Agilent Cary 630 FTIR, Waldbronn, Agilent Technologies, Santa Clara, CA, USA) equipped with an attenuated total reflectance accessory. The FTIR spectra of the conditioned samples (P_2_O_5_ for 1 week at 25 °C) were obtained at a resolution of 6 cm^−1^, in the wavelength range of 4000–650 cm^−1^, with 128 scans per spectrum. The analysis was performed in duplicate for each sample.

### 2.4. Reinforcing Capacity of the Microcrystalline Cellulose in PHBV Composite Films

#### 2.4.1. Production of the PHBV Films

PHBV films were produced to evaluate the reinforcing ability of MCCs obtained through the different mechanochemical methods. PHBV pellets were first dried at 60 °C and 0.8 mbar for 4 h to remove residual moisture. The dried PHBV pellets were then mixed with 1% of the different cellulosic samples and melt-blended in an internal mixer (Haake PolyLab QC, Thermo Fisher Scientific, Karlsruhe, Germany) at 170 °C and 50 rpm for 5 min. The resulting mixture was cold-ground with liquid nitrogen (Thermomix TM-5 homogeniser, Vorwerk Spain M.S.L., S.C., Madrid, Spain) for 20 s, and films were obtained by thermo-compressing 3.5 g of the respective powder in a hot-plate press (Model LP20, Labtech Engineering, Samut Prakan, Thailand) as follows: preheating for 5 min at 175 °C, thermos-compression at 175 °C and 100 bar for 4 min, and subsequent cooling for 3 min at 100 bar. Films without cellulose fractions were obtained as control formulation. All films were conditioned at 53% RH (supersaturated Mg(NO_3_)_2_ solution) at 25 °C for one week prior to characterisation.

#### 2.4.2. Characterisation of the Films

The optical properties of the films were evaluated in terms of colour and transparency using a spectrocolorimeter (CM-5, Konica Minolta Co., Tokyo, Japan) with a D65 illuminant and a 10° standard observer. Reflectance spectra were recorded between 400 and 700 nm using both white and black standard backgrounds. From these measurements, the internal transmittance and infinite reflectance were calculated using the Kubelka–Munk multiple-scattering theory. The transparency of each formulation was determined from its internal transmittance. The CIE L*a*b* colour parameters were obtained from the infinite reflectance spectra considering 10° observer and D65 illuminant. Chroma (C_ab_*) and hue angle (h_ab_*) were subsequently calculated from the a* and b* parameters, as well as the total colour difference (∆E*) with respect to the neat PHBV film. Measurements were performed at three different positions for each film, analysing three independent films per formulation.

The water vapour permeability (WVP) of the PHBV films was determined using a gravimetric method following ASTM E96/E96M, with modifications proposed by McHug et al. [30]. Conditioned film samples (3.5 cm diameter; 25 °C and 53% RH for one week) were sealed on Payne permeability cups (Elcometer SPRL, Hermalle-sous-Argenteau, Belgium) containing 5 mL of distilled water. The systems were placed in desiccators containing an oversaturated Mg(NO_3_)_2_ solution, maintained at 25 °C, and periodically weighed for 48 h. The WVP values were calculated from the slopes of the steady-state weight-loss versus time curves, considering the water vapour pressure gradient and the film thicknesses. The measurements were carried out in triplicate for each formulation.

An oxygen permeation analyser (Model 8101e, Systech Illinois, Johnsburg, IL, USA) was used to determine the oxygen permeability (OP) of the films according to ASTM D3985-05 [31]. The analyses were performed at 25 °C and 53% RH, and the oxygen transmission rate (OTR) was recorded until steady-state conditions were achieved. The measurements were performed in duplicate for each formulation.

The tensile properties of the films were determined according to ASTM D882 using a universal testing machine (TA.XTplus, Stable Micro Systems, Haslemere, UK). The conditioned film samples (25 mm × 100 mm; 25 °C and 53% RH for one week) were grabbed between two grips with an initial separation of 50 mm and stretched at a crosshead speed of 12.5 mm·min^−1^. From the stress–strain curves, the elastic modulus (EM), tensile strength at break (TS), and elongation at break (E) were determined. Eight replicates were analysed for each formulation.

The thermal stability of the films conditioned at 0% RH was analysed by TGA, as previously described for cellulosic samples. Likewise, phase transitions in the films were analysed by differential scanning calorimetry (DSC) using a Stare System analyser (Mettler-Toledo GmbH, Greifensee, Switzerland). Samples conditioned at 0% RH (5–7 mg) were sealed in aluminium pans and heated from −40 to 200 °C at a heating rate of 10 °C.min^−1^ under nitrogen flow (30 mL·min^−1^), maintained at 200 °C for 2 min, cooled from 200 to −40 °C (10 °C.min^−1^), held at this temperature for 2 min, and finally heated from −40 to 200 °C (10 °C.min^−1^). The analyses were carried out in duplicate.

The FTIR spectra of the different formulations were obtained as described in Section 2.3.7. The analysis was performed in duplicate for each formulation.

### 2.5. Statistical Analysis

The experimental data were analysed through analysis of variance (ANOVA) at a 95% confidence level using the Minitab Statistical Program (version 17). Significant differences (*p* < 0.05) were evaluated by applying Tukey’s studentised range (HSD) test. The results were reported as mean values ± standard deviation.

## 3. Results and Discussion

### 3.1. Process Yield

Figure 1 shows the flowchart of the global process for obtaining MCC from RS, including the first extraction step in subcritical water at 180 °C, to eliminate a great part of the non-cellulosic compounds, the second bleaching step with hydrogen peroxide, and the subsequent hydrolysis steps of the bleached fibres under different mechanochemical conditions. Table 1 presents the mass yields and chemical composition of the fibre (CF) recovery process. The raw RS contained 36.7% cellulose, 21.2% hemicellulose, 19.3% lignin, and 17% ash, in the previously reported range by other authors [8,32,33]. The SWE reduced the mass of the initial RS, giving rise to 75 g of LCF·100 g^−1^ RS, reflecting the removal of a great part of non-cellulosic compounds from the plant matrix, such as hemicellulose, proteins, lignin, waxes, phenolic compounds, or low-molecular-weight sugars [34]. This was confirmed by the chemical composition of LCF shown in Table 1. Considering the yield and composition of LCF, the estimated percentages of the structural components from the initial RS remaining in these fibres were 78, 10 and 83% of the total cellulose, hemicellulose and lignin, respectively. The reduction in the cellulose content can be attributed to its quantification method based on the glucose content, which overestimates cellulose, since part of glucose comes from the hemicellulose fraction, which is greatly hydrolysed and extracted during the SWE step [25]. Other authors also reported selective extraction of hemicellulose from different plant matrices when applying SWE in the range 160–180°C, yielding arabinoxylan oligomers [35,36,37]. The subsequent bleaching step yielded cellulose fibres with a high degree of purity (86.2 wt.%), while considering the mass yield and composition, only about 6% of lignin of the initial RS was maintained. Therefore, this step removes lignin from the LCF by the oxidation and cleavage of aromatic rings of its chemical structure [38]. This was reflected in the colour change observed between the LCF and CF samples, since conjugated aromatic groups act as chromophores responsible for the brownish colour of the LCF [39]. The ash content (mainly silica in RS) of the CF samples was also significantly reduced during delignification at pH 12, with an 88% decrease with respect to the RS content. This result is attributable to the solubilisation of amorphous silica in the alkaline medium, as also reported by other authors [40,41]. In RS, silica is mainly present as hydrated amorphous silica, including silanol-rich structures and polymerised silicic acid (polysilicic acid) weakly associated with the lignocellulosic matrix. These forms can be converted to soluble silicates under alkaline conditions and removed during the alkali bleaching, thereby reducing the ash content [42].

Table 2 shows the mass yields of MCC obtained using the different mechanochemical treatments applied to the CF, as well as the colour coordinates and whiteness index (*WI*) of all cellulosic samples. The mass yield expressed with respect to CF reflects the effectiveness of the hydrolysis treatments in partially removing the amorphous fraction of cellulose, while the yield expressed in terms of RS reflects the global efficiency of the overall green process in producing MCC from RS. In general, the mass yield of the hydrolytic process decreased as the reaction time increased for all treatments due to the greater progress of hydrolysis. This decrease was more pronounced for the high-shear homogenisation treatments (HS30 and HS60). This is consistent with the higher progress of hydrolysis of the amorphous fraction of CF and the corresponding release of hydrolysed molecules into the aqueous medium. The applied high-shear homogenisation facilitated the fibril disaggregation and enhanced acid diffusion into the fibril structure, promoting the preferential hydrolysis reaction, increasing the release of soluble cellulose-derived oligosaccharides, and, consequently, reducing the mass yield [43]. Specifically, the highest degree of hydrolysis was observed in the HS60 sample, which presented a global yield with respect to the RS of 27.9 g MCC.100 g^−1^ raw RS. Therefore, the more intense the mechanical treatment (time or shear), the greater the mass loss related to the partial removal of the amorphous matrix in the CFs and the greater the hydrolytic action of hydrochloric acid, resulting in greater chain cleavage and higher mass loss. Boschetti et al. [18] also reported lower yields of MCC and cellulose nanocrystals from eucalyptus pulp when the plant material was mechanically ground prior to acid hydrolysis. The authors also observed that a longer reaction time reduced the MCC yield.

The *WI* was used as a control variable for bleaching, as it is a good indicator of the cellulose purification process (Table 2). An increase in the *WI* value indicates greater removal of coloured compounds or greater alteration of the chromophore groups of the compounds present in the lignocellulosic matrix due to oxidation. The lightness (L*) of the CF sample increased significantly as a result of the delignification of the LCF, as previously discussed. Likewise, L* also showed a slight increase during the hydrolysis steps, with the increase being greater as the intensity of the treatment increased. This indicates that part of the residual lignin resistant to bleaching was also removed during acid hydrolysis, as also reported by Do et al. [44] for MCC samples obtained from different lignocellulosic wastes. Coherently, the colour saturation (C_ab_*) decreased after bleaching, but also in the progressively hydrolysed MCC, which was associated with small changes in the hue (Table 2). These changes in the optical properties of the cellulosic fractions reflected the loss of residual lignin and changes in the particle size that also affect the reflectance spectra of the powder.

### 3.2. Morphological and Microstructural Properties

**Figure 2:**
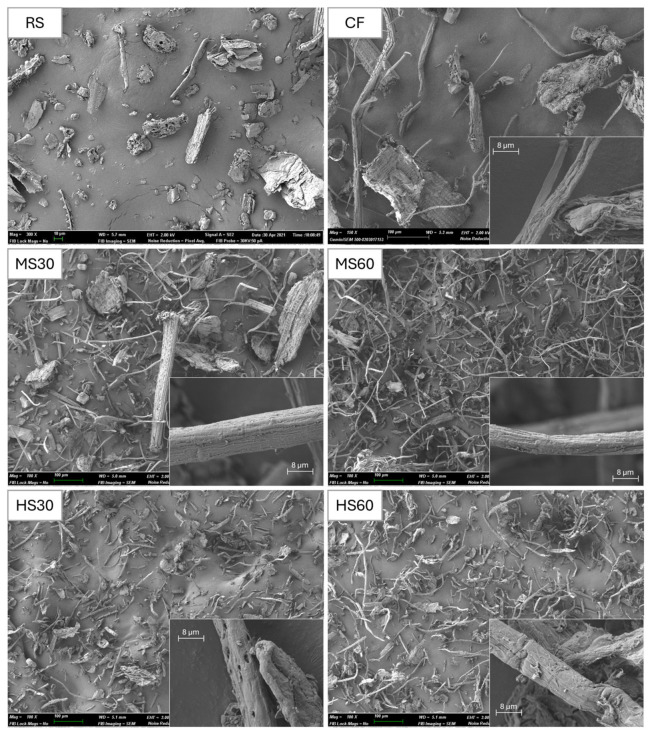
FESEM micrographs (×100) of untreated rice straw (RS), cellulose fibres (CF), and MCC obtained with different mechanochemical treatments: acid hydrolysis with mild stirring (MS30 and MS60) and high-shear homogenisation (HS30 and HS60) for 30 or 60 min. Micrographs of the CF and MCC at higher magnifications (×4000) were also included.

FESEM analysis was performed to analyse the microstructural changes in the RS matrix induced by SWE, the bleaching process, and the different mechanochemical hydrolysis to obtain MCC (Figure 2). The raw RS exhibited heterogeneous fibrous bundles of several dimensions (34 ± 20 µm and 12 ± 20 µm in length and diameter, respectively), resulting from the grinding process prior to extraction. These RS fragments exhibited a rod-like shape and compact configuration, revealing intact lignocellulosic tissue characterised by semicrystalline cellulose microfibrils embedded in a cementing matrix of lignin and hemicellulose [45]. After the combined cellulose purification process (SWE plus hydrogen peroxide bleaching), the CF showed partial defibrillation and a smoother and more homogeneous surface. This reflects the removal of non-cellulosic components from the lignocellulosic matrix, including lignin, hemicellulose, waxes, and silica, as reported by Othman et al. [46] for CF obtained from Semantan bamboo after the delignification step.

The application of different mechanochemical treatments resulted in the progressive disruption and defibrillation of the fibres, depending on the mechanical treatment and hydrolysis time. For treatments performed with mild stirring (MS30 and MS60), a greater degree of defibrillation of the cellulose fibre bundles was observed as the stirring time increased, in line with the greater progress of hydrolysis. Longer times promote the acid action due to the greater contact with the amorphous cellulose domains. In contrast, this progress with time was not so clearly observed in samples subjected to high-shear homogenisation, suggesting that mechanical defibrillation was effective from the short homogenisation times. After 30 min of high-shear treatment, the samples already showed a high degree of fragmentation and defibrillation, with shorter fibres than those obtained with MS treatments. This indicates the synergist effect of acid and mechanical action for producing MCC. Likewise, small voids distributed along the fibre surface can be observed in HS samples, which can be attributed to the hydrolysable matter removal by the hydrolytic action, as reported by Haafiz et al. [9] for MCC obtained from oil palm biomass residues. 

Therefore, FSEM micrographs clearly showed the acceleration of the acid hydrolysis by mechanical action of high shear, producing faster fragmentation of the CF and promoting the hydrolytic effect of acid. The shorter length of MCC facilitates handling and provides greater stiffness due to increased crystallinity, preventing fibre entanglement or agglomeration and resulting in more homogeneous dispersions.

**Figure 3:**
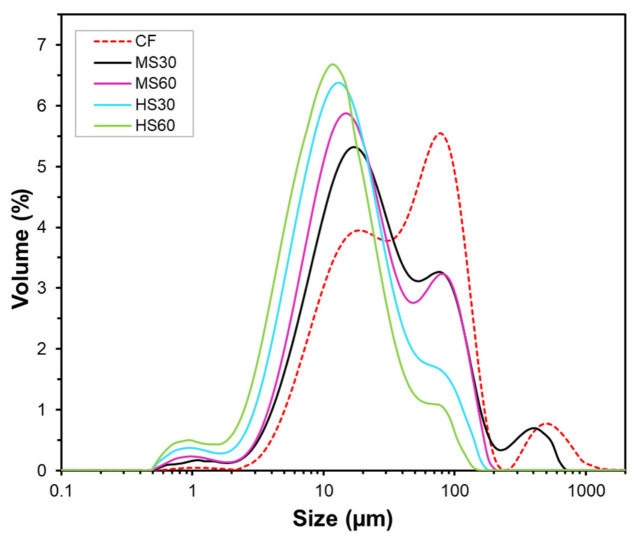
Particle size distribution from laser diffraction of cellulose fibres (CFs) and MCC samples subjected to different mechanochemical treatments with mild stirring (MS30, MS60) or high-shear homogenisation (HS30 and HS60) for 30 or 60 min.

The particle size distribution of the MCC samples was analysed using laser light scattering based on the Mie model. This model provides an exact solution to Maxwell’s equations for light scattering by spherical particles. Therefore, the determined sizes are related to the fibre length and the average radius of gyration of the equivalent sphere associated with the particle dispersed in the aqueous phase. The size distribution curves for the different cellulose samples are shown in Figure 3. In all cases, the curves exhibit main peaks around 10 and 100 µm, indicating bimodal distributions. The non-hydrolysed bleached fibres (CFs) and the MCC treated with lower treatment intensity (MS30) exhibit a slight peak with a higher proportion of particles of approximately 500 µm, suggesting the presence of large fragments. This peak disappears as the hydrolysis progressed in the S60 and HS samples. Therefore, the hydrolysis, greatly enhanced by the high shear, contributed to reducing the particle size of the biggest particles. Likewise, the volume fraction of the population of about 100 µm was strongly reduced in the HS treatments, while the volume fraction of shorter particles increased, and a new small peak appeared at about 1 µm, thus reflecting the effectiveness of the homogenisation for the defibrillation and breaking of cellulose fibres. The mean particle size of MCC obtained from different plant-based residues ranged between 10 and 100 µm, as reported by Ventura-Cruz et al. [6]. This range included the MCCs obtained from RS with the applied treatments, mainly samples HS30 and HS60. It is noteworthy that the high-shear homogenised fibres HS30 and HS60 exhibited a practically monomodal distribution, with a peak at 10 µm after 30 min of treatment. Therefore, the effect of time was not as significant as the mechanical effect on the size distribution, as also observed in the FESEM images (Figure 2). The MCC samples obtained by high shear had a similar particle size as the MCC industrially produced from bleached Kraft wood fibres and sulfuric acid treatment (10–50 µm in diameter) [47].

### 3.3. Intrinsic Viscosity, Degree of Polymerisation, and Molecular Weight

Table 3 summarises the intrinsic viscosity ([Ƞ]) and the related parameters degree of polymerisation (DP) and viscous average molecular weight (Mv) for the different cellulosic samples. The bleached fibres (CF) presented [η], DP, and Mv values similar to those found for spruce cellulose fibres [28], while the DP was higher than that reported for crude cellulose from rice straw purified by alkaline and H_2_O_2_ treatments, with and without microwave assistance [21]. A sharp decrease in the three parameters was observed for MCC, provoked by hydrolysis treatments, which promote the breakdown of glycosidic bonds and a progressive decrease in cellulose molecular weight. The obtained values were in the range of those previously reported for MCC obtained from rice straw [20,21]. The reduction in these parameters was higher as the severity of the hydrolysis treatment intensified, whether by extending the treatment time or by applying high-shear homogenisation. Compared to the bleached CF, the less hydrolysed MCC sample (MS30) showed a reduction of approximately 51%, 55%, and 53%, respectively, in [η], DP, and Mv values. This reduction became more pronounced with longer hydrolysis times and the application of high-shear homogenisation. In general, acid hydrolysis of native cellulose occurs faster to the so-called level-off DP (LODP), from which DP decreases much more slowly. The LODP has been correlated with crystal sizes along the original cellulose chains before the acid hydrolysis based on the hypothesis of a regular distribution of disordered or para-crystalline domains along the microfibres [48]. The high-shear treatment accelerated the hydrolytic cleavage of the amorphous regions to the LODP, probably due to its defibrillation effect that implies a greater exposure of the amorphous domains to the acid attack. Therefore, the combination of mechanical and chemical treatments enhanced the acid cleavage by increasing fibre accessibility and disrupting the fibre structure, thereby reducing DP values faster. The effectiveness of mechanical treatment was also observed by Thielemans et al. [49], who obtained short-polymer microcrystalline cellulose (DP below 70) by first decreasing the LODP with an intensive ball mill treatment of MCC, followed by mild acid hydrolysis. 

The HS60 MCC sample presented the lowest [η] and DP values, which was in the range reported for commercial Avicel, a colloidal cellulose used in many applications, which has DP values ranging from 200 to 400, depending on the grade. This suggests that the combination of hydrolysis and high-shear homogenisation was very effective in producing highly depolymerised MCC similar to commercial samples with only 30–60 min of acid treatment.

### 3.4. Crystallinity Analysis

**Figure 4:**
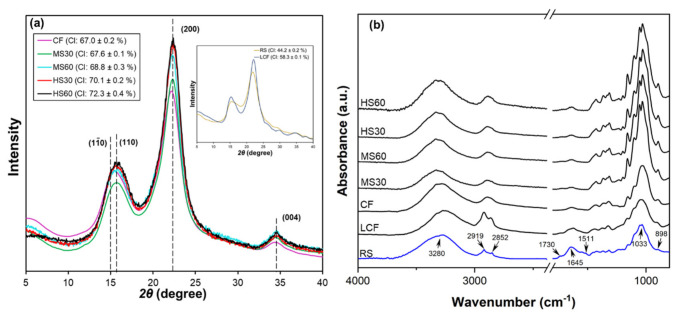
XRD (**a**) and FTIR (**b**) spectra of rice straw (RS) and cellulosic fibres subjected to different treatments: subcritical water extraction (LCF) plus bleaching (CF) and acid hydrolysis with magnetic stirring (MS30 and MS60) and high-shear homogenisation (HS30 and HS60) for 30 or 60 min. The crystallinity index (CI) of all samples, as well as the XRD spectra of the RS and LCF fractions (inserted graph), are included for comparison purposes (**a**).

The degree of crystallinity of the different cellulosic samples was investigated through X-ray diffraction (XRD). Figure 4a shows the diffraction patterns and crystallinity index (CI) of bleached CF and MCC samples obtained with different mechanochemical treatments, as well as the XRD spectra and CI values of RS and the extraction residue (LCF) (inserted graph). All the cellulosic samples exhibited a type Iβ cellulose diffraction pattern, characterised by the presence of peaks at 2*θ* = 15° (1$\bar{1}$0), 16° (110), 22° (200), and 34° (004). This result indicates that the allomorphic form of cellulose found in nature was preserved in the MCC, as reported by other authors [50,51].

The XRD diffractograms of the RS, LCF, and CF confirmed the effectiveness of the combined method (SWE followed by H_2_O_2_ bleaching) to purify cellulose from RS. A notable increase in the CI was observed after SWE (LCF, CI: 58%) compared to RS (CI: 44%), which is attributable to the selective removal of amorphous components from the plant matrix, especially hemicellulose (Table 2). Compared to the LCF sample, the increase in the CI value of the bleached CF (67%) is associated with the further purification of cellulose during bleaching, which removes non-cellulosic compounds, mainly lignin, as also reported by Freitas et al. [8]. Ibrahim et al. [20] reported CI values of 65% for fibres of rice straw bleached with sodium hypochlorite previously processed by alkaline–acid or acid–alkaline pulping.

As expected, an increase in CI was observed for the MCC samples, which became more pronounced as the intensity of the mechanochemical treatment rose, coherently with the increasing removal of amorphous regions from the fibre structure. Furthermore, the improvement in cellulose purification, deduced from the *WI* increase, may also have contributed to the enhancement of the sample crystallinity. The HS60 sample exhibited the highest CI (72%), coherently with the higher hydrolytic cleavage of glycosidic bonds during hydrolysis, enhanced by high-shear homogenisation and the subsequent solubilisation of hydrolysed amorphous regions. Therefore, high-shear homogenisation greatly promoted the particle size reduction, defibrillation and acid attack, favouring the production of more crystalline MCC with shorter treatment times. In contrast, the samples from the MS30 treatment exhibited CI values nearer to those of the bleached fibres. The values of the CI of MCC obtained from different lignocellulosic residues ranged between 62% and 92%, depending of the origin and treatments applied [6]. Wistara et al. [22] reported CI values of 68% for rice straw, 87% for soda pulp, and 80% for MCC. For the MCC obtained from RS fibres by enzyme hydrolysis, CI values of 67 and 82% were reported, depending on the previous process of cellulose purification [20]. Therefore, HS30 and HS60 samples can be considered as notably crystalline MCCs.

### 3.5. Fourier-Transformed Infrared Spectroscopy (FTIR)

Figure 4b shows the FTIR spectra of the cellulosic samples obtained at the different steps of the cellulose purification process. In all cases, a bell-shaped absorption band was observed at approximately 3280 cm^−1^, corresponding to the O-H stretching vibration involved in hydrogen bonding [7]. In the raw RS, this band is broader, indicating a greater presence of hydrogen bonds and greater structural heterogeneity, mainly attributable to the presence of hemicellulose and lignin. In contrast, this band is more defined in the purified samples, reflecting an increase in cellulose content.

The absorption bands at 2919 and 2852 cm^−1^, observed in all samples, are attributable to the C-H asymmetric and symmetric stretching vibrations present in the structure of cellulose, hemicellulose, and lignin [52]. These peaks were more attenuated in the bleached samples than in the RS and LCF, reflecting a lower contribution of C-H vibration to the spectra due to the cellulose purification. The peak at 1730 cm^−1^ corresponds to the C=O stretching vibration of ester and carboxylic groups associated with hemicellulose and lignin fractions [45]. This band was clearly observed in the RS sample and progressively decreased until it disappeared in the treated samples, thus confirming the removal of these compounds during the purification process. A similar trend was observed for the band at 1511 cm^−1^, which is associated with the C=C stretching vibration of aromatic rings present in the lignin structure [53]. The peak at 1645 cm^−1^, assigned to the O-H bending vibration of adsorbed water, was observed in all samples and decreased as the cellulose purification progressed and crystallinity increased, reflecting the lower moisture absorption capacity of the more crystalline material [45]. All samples exhibited a band at 1033 cm^−1^, attributed to the C-O-C stretching vibration of the glycosidic ring in the cellulose backbone. This band became sharper and more defined in the treated samples, reflecting the high degree of cellulose purification, as observed in previous studies of cellulose purification from RS [8]. A similar pattern was observed for the band at 898 cm^−1^, assigned to the β-glycosidic linkage, considered a characteristic marker of type I cellulose that is the typical crystalline structure of plant-derived cellulose. This peak became more pronounced in the MCC samples and increased when the hydrolysis progressed. The FTIR spectra thus demonstrate the effectiveness of the cellulose purification process used in this study, with the HS60 treatment standing out for its ability to produce a material with a high degree of cellulose purity, although the FTIR spectra were not highly sensitive to changes in the cellulose crystallinity.

### 3.6. Thermal Stability

**Figure 5:**
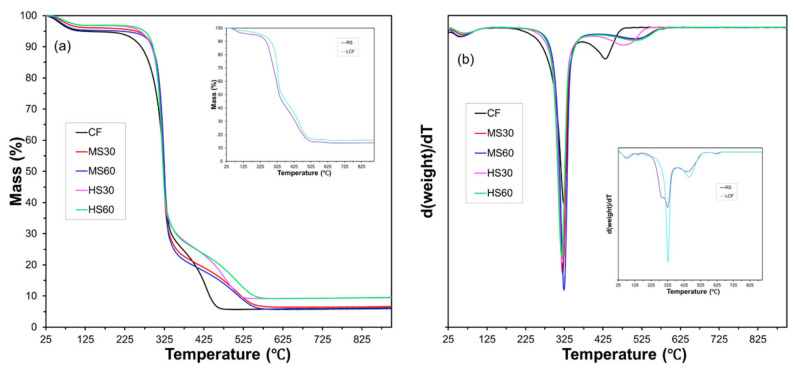
TGA curves of the percentage of mass loss as a function of temperature (**a**) and its first derivative (DTGA) curves (**b**) of cellulosic samples subjected to different treatments: SWE plus bleaching (CF) and acid hydrolysis with agitation (MS30 and MS60) and high-shear homogenisation (HS30 and HS60) for 30 or 60 min. The thermograms and DTGA curves of the RS and LCF samples are included for comparison purposes (inserted graphs).

The thermal stability of the samples was analysed by TGA to assess the effects of the homogenisation method and reaction time on the thermal degradation of the MCC. Figure 5 shows the TGA and DTGA curves for the different cellulose samples. All thermograms showed an initial weight-loss stage between 125 and 130 °C, corresponding to the loss of bound water, which accounted for approximately 4–5% of the initial mass. This mass loss was similar for all samples, suggesting similar levels of bound water content after the conditioning at 0% RH.

A marked weight loss was observed in all samples at temperatures ranging from 200 to 375 °C, corresponding to the typical thermodegradation pattern of lignocellulosic samples with overlapped weight loss of their components (hemicellulose, cellulose and lignin) present in different proportions. As previously described [54,55,56], degradation of hemicellulose occurs between 150 and 350 °C, cellulose between 275 and 350 °C and lignin between 250 and 500 °C. The lowest onset degradation temperature was for the RS samples, followed by the LCF sample, bleached CF, and the different MCC samples (MS30, MS60, HS30, and HS60). This behaviour reflects the progressive removal of hemicellulose during the successive treatments of RS, thereby shifting the degradation temperature towards a higher temperature characteristic of pure cellulose. Hideno [57] reported a similar increase in thermostability after the removal of amorphous components in chemically treated lignocellulosic biomass. The CF and MCC samples exhibited similar values of temperature at maximum degradation rates (323–325 °C), but the CF sample showed a broader, less defined peak in the DTGA curve, which reflects its lower structural homogeneity, with the presence of residual hemicellulose and more amorphous cellulose in the sample. In contrast, the MCC samples exhibited more intense, well-defined peaks, indicating a higher level of cellulose purity and crystallinity.

Likewise, compared to the MCC samples, the CF exhibited greater mass loss in the temperature range of 375–600 °C, attributed to degradation of residual lignin. Therefore, the hydrolysis treatments also contribute to removal of the residual lignin in the samples, as also deduced from the progressive increase in WI of the samples (Section 3.1). Therefore, the greater thermal stability of MCCs, reflected by the shift of the main degradation peak to higher temperatures and its narrowing in DTGA curves, is consistent with the increase in cellulose purity and crystallinity.

### 3.7. Reinforcing Properties in Composite PHBV Films

MCC is a highly promising renewable filler option for polymer matrices due to its exceptional mechanical strength, lightweight properties, stiffness, and high aspect ratio, allowing considerable improvements in tensile and flexural properties with low filler loading (0.5–2%), while it adheres to sustainability objectives in both its sourcing and disposal [58,59]. The effect of incorporating the different cellulosic fillers, including CF and MCC obtained through different mechanochemical treatments, on the functional properties of PHBV films was analysed in order to determine the influence of the sample properties on their reinforcing effect. This effect is related to the force transfer mechanisms between the dispersed phase and the polymer matrix, which depends on the nature of filler–polymer interactions and the degree of filler dispersion [47]. Table 4 shows the physical properties characterised in PHBV films and composites with 1%wt. of the different RS cellulosic fillers obtained (CF and different MCC).

#### 3.7.1. Physical Properties of the Composites

The incorporation of CF or MCCs did not significantly affect the appearance of the composite films compared to the neat PHBV formulation, as shown in Table 4. This was supported by the slight differences in the colour parameters between the films, which exhibited similar lightness (L*), colour saturation (C_ab_*) and hue (h_ab_*) values. Likewise, the small values in total colour difference (∆E*) with respect the neat PHBV are in the range of non-perceptible values. Moreover, all formulations exhibited similar transparency, as reflected in both the images and the transmittance values at 500 nm (Table 4). Other authors also reported well-dispersed cellulosic fillers within the polymer matrix, resulting in composite films with no appreciable changes in appearance and colour with respect to the neat polymer films [60,61,62].

The water vapour (WVP) and oxygen (OP) permeabilities of the films are also shown in Table 4. The neat PHBV film exhibited WVP and OP values of 5.5 × 10^−12^ g.Pa^−1^.s^−1^.m^−1^ and 3.0 × 10^−13^ cm^3^.m^−1^.s^−1^.Pa^−1^, respectively, as reported by other authors [63]. Nevertheless, the presence of cellulosic reinforcement affected the barrier properties of the composite films, depending on the properties of the filler. In general, the incorporation of MCC slightly reduced both WVP and OP, whereas the CF-containing composite films exhibited values closer to those found for the control formulation. The MCC–composite films exhibited a progressive decrease in water vapour permeability as the hydrolysis treatment of cellulose intensified, with reductions in WVP of 29%, 34%, 45%, and 53% for the films containing MS30, MS60, HS30, and HS60, respectively (*p* < 0.05). Similar levels of reduction in WVP were reported by other authors when MCC was incorporated into a polyvinyl alcohol/carboxymethyl cellulose matrix [64]. The MCC was less effective in reducing OP, with films containing HS30 and HS60 being the least permeable, showing an OP reduction of approximately 17% (*p* < 0.05). So, the effectiveness of the filler at improving the barrier capacity of the composites was correlated with the reduction in their particle size and the increase in crystallinity. The improvement in the barrier properties of MCC-containing films was associated with the higher crystallinity of MCC and the good dispersion and entanglement of the cellulosic fillers within the PHBV matrix. This leads to a reduction in the free volume of the polymer network whilst increasing the tortuosity factor, hindering the diffusion of small molecules, such as water and oxygen, through the polymer matrix [65]. The structural differences between CF and MCC are reflected on their different mechanical and barrier effects on the polymer, with the MCC being more effective as a reinforcing or barrier agent when its crystallinity increases and particle size decreases. In this sense, Bandera et al. [16] obtained cellulose whisker-like materials by mechanical treatment of commercially available MCC, which exhibited a similar reinforcing effect to that of cellulose whiskers.

The mechanical performance of films based on PHBV containing different cellulosic fillers was evaluated in terms of tensile strength at break (TS), elongation at break (E), and the elastic modulus (EM) (Table 4). The incorporation of fillers significantly affected the tensile properties of the PHBV films, particularly for the formulations containing HS30 and HS60. Both MCCs, obtained with high-shear homogenisation, gave rise to films more resistant to break (~14%) and that were more stretchable (~50%), while the HS60 sample also slightly increased the stiffness of the composite film (~6%). Other authors also reported improvements in the tensile properties of cellulose-reinforced PHBV films, in which the fillers exhibited good dispersion and interfacial adhesion with the polymer matrix [66,67]. This behaviour is attributable to the greater reinforcing capacity of the highly crystalline MCC, which has high stiffness and mechanical strength, thereby transferring its strength to the PHBV matrix. Therefore, the higher crystallinity, smaller particle size and lower DP of HS-MCC improved their dispersibility, compatibility, and interfacial adhesion within the polymer matrix, exhibiting a more similar reinforcing effect to that of nanocellulose structures [16].

#### 3.7.2. FTIR Spectra

**Figure 6:**
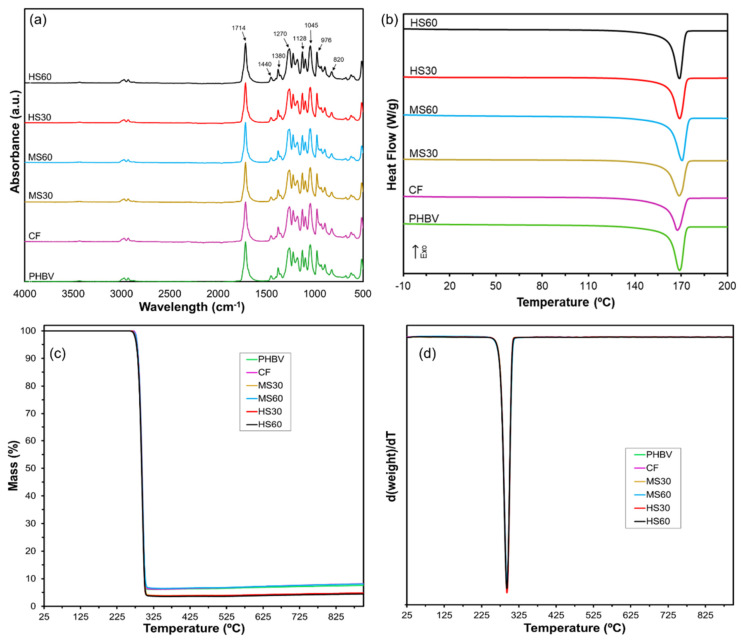
FTIR spectra (**a**), second heating DSC thermograms (**b**), and TGA (**c**) and DTGA (**d**) curves of the PHBV films with or without different cellulosic samples, typically bleached CF and MCC obtained with different mechanochemical treatments (MS30, MS60, HS30, and HS60), at 1%wt.

Figure 6a shows the vibrational profile of the functional groups present in the PHBV films, both in the control and in those containing different cellulosic fillers. The FTIR spectra of the PHBV film showed no significant changes after the incorporation of cellulosic samples. The typical FTIR spectrum pattern of PHBV films was observed, characterised by absorption bands at: 3000–2930, 1270, and 1220 cm^−1^ (C-H stretching vibrations); 1714 cm^−1^ (C=O stretching vibration); 1440 and 1380 cm^−1^ (C-H bending vibrations); 1185, 1128, and 1045 cm^−1^ (C-O stretching vibrations); and 820–976 cm^−1^ (C-H stretching vibrations) [63]. The maintenance of these bands in the composite films suggests that no new chemical bonds were formed and that the interaction between the PHBV matrix and the cellulose fillers is mainly physical in nature, as also reported by other authors [68]. It is worth mentioning that the low concentration of CF and MCC in the films did not allow for the detection of characteristic cellulose bands, which were masked by peaks from the predominant PHBV matrix.

#### 3.7.3. Thermal Behaviour

The thermal behaviour of PHBV films containing CF and different MCCs was investigated by DSC and TGA. Figure 6b shows the DSC curves of the second heating, while Table 5 summarises the thermal parameters obtained from the first and second heating, as well as in the cooling step. In general, these parameters, including the glass transition temperature (T_g_), melting temperature (T_m_) and enthalpy (ΔH_m_), crystallisation temperature (T_c_), and degree of crystallinity (X_c_), remained almost unchanged (T_m1_ and T_m2_ ~ 168–170 °C, X_c_ ~ 69–74%, T_c_ ~ 121–122 °C). This suggests that the incorporation of CF and MCC did not affect the final crystalline structure or the crystallisation behaviour of PHBV at the concentration used (1%wt.). Although cellulose and its derivatives have been reported as heterogeneous nucleating agents in the PHBV matrix [21,69], promoting an increase in T_c_ and accelerating crystallisation, this effect is highly dependent on the polymer–filler interfacial interactions, the dispersion degree, and the morphology and concentration of the filler. Mathel et al. [66] reported that the nucleating effect of cellulose in PHBV is not always significant and depends on filler characteristics and dispersion.

The thermal stability of the films, analysed by TGA, was not affected by the presence of cellulose fillers, whether CF or MCC. All samples exhibited a similar degradation pattern, with a single main thermal event between 255 and 315 °C, characteristic of PHBV [63]. Similar results have been reported for PHBV films reinforced with cellulose [70].

## 4. Conclusions

The RS cellulose fibres obtained by combining subcritical water extraction and bleaching with H_2_O_2_ exhibited high cellulose purity (86%) and crystallinity (67%), with a 35% mass yield relative to the raw material, reducing the usage of chemicals in the process. Hydrolysis with HCl 2N at 60 °C yielded MCCs with varying particle sizes, crystallinity (68–72%), and degrees of polymerisation (627–371) depending on the treatment time and mechanical agitation. High-shear homogenisation greatly accelerated the hydrolysis process of the amorphous fraction of the fibres, contributing to the reduction in particle size (to about 10 µm in mean diameter), defibration of the initial fibres, and increased crystallinity (70–72%). After 30–60 min of treatment, the resulting MCCs exhibited properties similar to commercial AVICEL, with a greater reinforcing capacity and barrier effect in the PHBV composite films than those obtained with mild agitation. Therefore, the proposed process to obtain MCC from rice straw constitutes an alternative to producing cellulose materials with functional properties close to the nanocellulose but more scalable and sustainable than the usual methods.

## Figures and Tables

**Table 1:** Mass yield (with respect to the RS mass) and content of structural components (cellulose, hemicellulose, lignin, and ashes) in RS and lignocellulosic fibres at the different steps of the isolation process.

Sample	Yield (%wt.)	Cellulose (%wt.)	Hemicellulose (%wt.)	Klason Lignin (%wt.)	Ashes (%wt.)
RS	-	36.7 ± 0.4	19.3 ± 0.1	21.2 ± 0.5	17 ± 2
LCF	75.0	38.1 ± 2.0	2.7 ± 0.1	23.7 ± 1.5	13 ± 5
CF	35.4	86.2 ± 0.2	1.5 ± 0.6	3.4 ± 0.1	6 ± 1

**Table 2:** Mass yields of the different cellulosic samples with respect to the bleached cellulose fibres (CF) and rice straw (RS), and colour coordinates (L*, C* and h*) and whiteness index (*WI*) (mean ± standard deviation).

Sample	Yield (g MCC. 100 g^−1^ CF)	Yield (g MCC. 100 g^−1^ RS)	L*	C*	h*	*WI*
RS	-	-	56.4 ± 0.1	22.2 ± 0.1	76.8 ± 0.1	51.1 ± 0.1
LCF	-	-	35.2 ± 0.2	17.3 ± 0.3	69.0 ± 0.1	33.0 ± 0.2
CF	-	-	87.7 ± 0.2	15.6 ± 0.1	90.5 ± 0.3	80.1 ± 0.1
MS30	93.3	32.6	87.6 ± 0.1	9.4 ± 0.1	86.4 ± 0.1	85.7 ± 0.2
MS60	89.8	31.3	88.4 ± 0.1	9.5 ± 0.2	86.3 ± 0.1	85.6 ± 0.1
HS30	83.7	29.2	88.1 ± 0.8	9.2 ± 0.1	87.5 ± 0.3	87.0 ± 0.7
HS60	79.8	27.8	90.7 ± 0.7	9.1 ± 0.1	87.1 ± 0.2	90.4 ± 0.8

**Table 3:** Intrinsic viscosity ([Ƞ]), degree of polymerisation (DP) and molecular weight (M_v_) of different cellulosic samples subjected to different treatments: SWE plus bleaching (S180) and acid hydrolysis with magnetic stirring (MS30 and MS60) and high-shear homogenisation (HS30 and HS60) for 30 or 60 min.

Sample	[Ƞ] (mL.g^−1^)	DP	M_v_ (g.mol^−1^)
CF	409 ± 9	1380 ± 33	243,000 ± 6000
MS30	201 ± 1 ^a^	627 ± 3 ^a^	115,200 ± 600 ^a^
MS60	185 ± 2 ^b^	573 ± 5 ^b^	106,100 ± 900 ^b^
HS30	161 ± 1 ^c^	492 ± 4 ^c^	92,400 ± 700 ^c^
HS60	125 ± 12 ^d^	371 ± 38 ^d^	72,000 ± 6000 ^d^
MCC, Avicel^®^ *	128	380	61,600
Spruce sulphite pulp *	435	1480	240,000

* Values from Gericke et al. [28]. Different subscript letters in the same column for MCC samples indicate significant differences according to the Tukey test (α = 0.05).

**Table 4:** Appearance, optical properties (L*, C*, h*, ∆E*, and transmittance at 500 nm (*T*_500_)), barrier (WVP and OP), and tensile properties (TS, E, and EM) of the PHBV films with or without different cellulosic samples, typically bleached CF and MCC obtained with different mechanochemical treatments (MS30, MS60, HS30, and HS60), at 1%wt. (mean ± standard deviation).

Property	PHBV	CF	MS30	MS60	HS30	HS60
Appearance	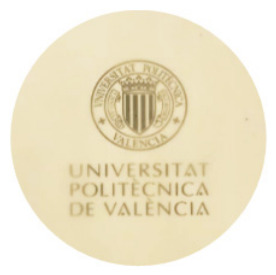	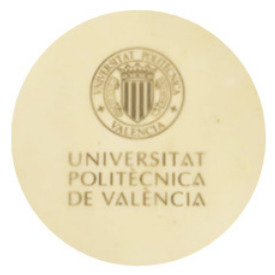	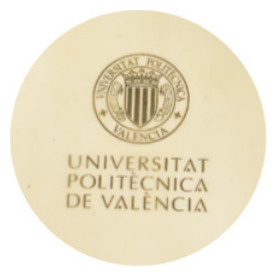	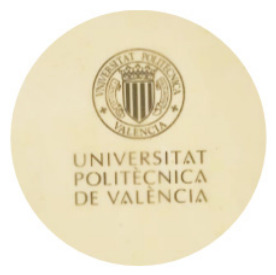	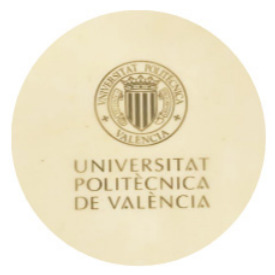	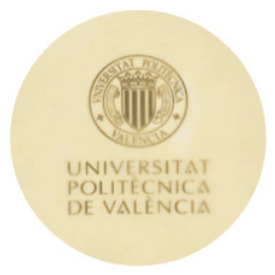
L*	73.0 ± 1.0 ^a^	72.0 ± 0.2 ^a^	72.0 ± 0.2 ^a^	72.3 ± 0.3 ^a^	71.0 ± 0.6 ^b^	72.0 ± 0.5 ^ab^
C*	17.6 ± 0.1 ^b^	19.0 ± 0.6 ^a^	19.1 ± 0.5 ^a^	19.0 ± 0.8 ^a^	19.6 ± 0.8 ^a^	19.0 ± 0.3 ^a^
h*	78.1 ± 0.3 ^a^	77.1 ± 0.1 ^b^	76.5 ± 0.1 ^b^	77.3 ± 0.1 ^b^	76.2 ± 0.4 ^ab^	77.2 ± 0.2 ^b^
∆E*	-	2.0 ± 0.6	2.2 ± 0.4	1.4 ± 0.8	2.0 ± 1.0	1.6 ± 0.5
T_500_	0.53 ± 0.02 ^a^	0.54 ± 0.02 ^a^	0.54 ± 0.01 ^a^	0.54 ± 0.03 ^a^	0.53 ± 0.01 ^a^	0.54 ± 0.01 ^a^
WVP × 10^12^ (g.Pa^−1^.s^−1^.m^−1^)	5.5 ± 0.4 ^a^	4.4 ± 0.1 ^b^	3.9 ± 0.2 ^c^	3.6 ± 0.2 ^c^	3.0 ± 0.2 ^d^	2.6 ± 0.1 ^e^
OP × 10^13^ (cm^3^.m^−1^.s^−1^.Pa^−1^)	3.0 ± 0.2 ^b^	3.6 ± 0.1 ^a^	2.9 ± 0.4 ^bc^	3.3 ± 0.2 ^b^	2.7 ± 0.2 ^c^	2.5 ± 0.2 ^c^
TS (MPa)	35.4 ± 2.0 ^b^	34.2 ± 1.9 ^b^	34.0 ± 1.6 ^b^	35.0 ± 1.1 ^b^	39.1 ± 1.9 ^a^	41.6 ± 3.7 ^a^
E (%)	1.9 ± 0.1 ^b^	2.1 ± 0.2 ^b^	2.2 ± 0.2 ^b^	2.2 ± 0.2 ^b^	2.9 ± 0.4 ^a^	2.8 ± 0.5 ^a^
EM (MPa)	1863 ± 45 ^b^	1792 ± 82 ^b^	1856 ± 23 ^b^	1828 ± 44 ^b^	1779 ± 63 ^b^	1981 ± 55 ^a^

Different letters in the same row indicate significant differences between films by the Tukey test (α = 0.05).

**Table 5:** DSC parameters (glass transition temperature (T_g_), melting temperature (T_m_), melting enthalpy (ΔH_m_), and degree of crystallinity (X_c_)) from the first and second heating and crystallisation temperature (T_c_) from the cooling step.

	First Heating				Cooling	Second Heating		
Film	T_g_ (°C)	T_m1_ (°C)	∆H_m1_ (J.g^−1^ PHBV)	X_c_ (%)	Tc (°C)	T_m2_ (°C)	∆H_m2_ (J.g^−1^ PHBV)	X_c_ (%)
PHBV	6.8 ± 0.5	170.0 ± 0.5	90.8 ± 4.8	68.8 ± 1.7	122.8 ± 2.4	168.4 ± 0.7	97.1 ± 3.4	73.5 ± 2.6
CF	6.5 ± 0.1	169.8 ± 0.4	88.6 ± 2.5	64.8 ± 5.4	119.8 ± 1.1	167.5 ± 2.0	91.4 ± 3.6	69.3 ± 2.7
MS30	6.2 ± 0.6	169.8 ± 3.1	85.5 ± 3.1	64.7 ± 2.4	121.2 ± 1.0	168.8 ± 2.0	91.7 ± 0.8	69.5 ± 0.6
MS60	6.3 ± 0.6	169.8 ± 0.8	91.5 ± 2.8	69.3 ± 2.1	121.6 ± 0.3	169.7 ± 0.2	99.9 ± 3.1	73.9 ± 0.1
HS30	7.1 ± 0.1	170.6 ± 0.6	85.9 ± 0.3	65.1 ± 0.2	121.7 ± 0.7	168.6 ± 0.3	95.0 ± 0.3	72.0 ± 0.2
HS60	6.9 ± 0.3	171.5 ± 0.5	87.7 ± 0.7	66.5 ± 0.5	122.0 ± 0.1	168.3 ± 0.3	95.2 ± 1.0	72.1 ± 0.8

## Data Availability

Data are contained within this article.
